# Resilience in Children: Developmental Perspectives

**DOI:** 10.3390/children5070098

**Published:** 2018-07-17

**Authors:** Ann S. Masten, Andrew J. Barnes

**Affiliations:** 1 Institute of Child Development, University of Minnesota Twin Cities, Minneapolis, MN 55455, USA; 2 Developmental-Behavioral Pediatrics, Division of General Pediatrics and Adolescent Health, Department of Pediatrics, University of Minnesota Medical School, Minneapolis, MN 55455, USA; drbarnes@umn.edu

**Keywords:** resilience, stress, risk, vulnerability, system, protective factor, pathways, cascade

## Abstract

Advances in developmental resilience science are highlighted with commentary on implications for pediatric systems that aspire to promote healthy development over the life course. Resilience science is surging along with growing concerns about the consequences of adverse childhood experiences on lifelong development. Resilience is defined as the capacity of a system to adapt successfully to challenges that threaten the function, survival, or future development of the system. This definition is scalable across system levels and across disciplines, applicable to resilience in a person, a family, a health care system, a community, an economy, or other systems. Robust findings on resilience in childhood underscore the importance of exposure dose; fundamental adaptive systems embedded in the lives of individuals and their interactions with other systems; developmental timing; and the crucial role of healthcare practitioners and educators as well as family caregivers in nurturing resilience on the “front lines” of lived childhood experience. Resilience science suggests that human resilience is common, dynamic, generated through myriad interactions of multiple systems from the biological to the sociocultural, and mutable given strategic targeting and timing. Implications for pediatric practice and training are discussed.

## 1. Introduction

Evidence continues to accumulate on the short- and long-term risks to health and well-being posed by adverse life experiences in children, particularly when adversities are prolonged, cumulative, or occurring during sensitive periods in early neurobiological development [1,2,3,4,5]. At the same time, there is growing concern about the impact of disasters, war, poverty, pandemics, climate change, and associated displacement on the global well-being of children [6]. This confluence of threats to the present and future health of children and, concomitantly, their societies may be motivating a surge of global interest in resilience that spans multiple sectors and sciences [7,8,9,10,11,12]. Attention to resilience science and its implications for practice also is rising in pediatric research and practice [5]. The purpose of this article is to highlight the meaning and significance of an integrated and developmental systems perspective on resilience in human development, with particular implications for pediatric research, practice, and training.

### 1.1. Changing Definitions of Resilience

Resilience science emerged in research on children around 1970 as pioneering investigators studying children at risk for psychopathology and related problems recognized the significance of the striking variability they were observing among groups of children contending with multiple risks and adversities [12]. In the ensuing five decades, investigators in multiple disciplines, most notably in psychology, psychiatry, pediatrics, and education, pursued the goal of explaining this observable variation. They aimed to understand positive developmental trajectories among children who managed to hold their own or even flourish despite adversity and, ultimately, to learn how to promote healthy development among children threatened by adverse childhood experiences.

Patterns of positive development in the context of adversity were broadly described as “resilience” phenomena, although investigators varied in their definitions of resilience as referring to capacity, processes, or outcomes of positive adaptation to adversity [13]. Variable definitions have plagued this literature in multiple disciplines over the years, creating challenges for systematic reviews and meta-analyses of the findings [14,15]. Nonetheless, there are some remarkable consistencies in findings from the voluminous literature on resilience, discussed further below.

Over the past two decades, a shift has occurred in the definition of resilience, likely driven by the growing dominance of developmental systems theory (DST) as the central theory in developmental science [6,8,12,16,17,18]. Development of an individual in this framework emerges from myriad interactions across system levels, shaped by the interplay of processes within and between individuals and their contexts, at multiple levels of function from the molecular to the macro-level systems of culture, society, and ecology. As a result, development is probabilistic, dynamic, nonlinear, and shaped by processes integrating many systems. It is a transdisciplinary framework that is revolutionizing theory and research, and more gradually, practice and policy.

In the domain of health, the emerging framework of “life course health development” (LCHD) similarly reflects the fundamental shift from a disease-oriented framework to a more comprehensive, developmental, and dynamic health-oriented approach [19]. LCHD also is grounded in DST.

Masten and her colleagues have argued that unifying evidence from different sciences concerned with resilience across disciplines and sectors is essential for adequate research, preparation, intervention, and recovery efforts in disasters and other multi-system calamities, including terror attacks or pandemics [6,12,15,17,20,21,22]. Global threats require integrated theories, knowledge, intervention, and training, all of which are facilitated by scalable definitions that can be aligned across sectors and disciplines [6].

### 1.2. Definition of Resilience from a Developmental Systems Perspective

Resilience can be broadly defined as the capacity of a system to adapt successfully to challenges that threaten the function, survival, or future development of the system [6,12,17]. This definition is intended to be scalable across system levels and portable across disciplines. Resilience is a feature of complex adaptive systems, including human individuals, but also families, economies, ecosystems, and organizations. This definition can also be applied to systems within an individual, such as the human immune system.

One of the most important implications of this definition is the idea that the resilience of a developing person is not circumscribed within the body and mind of that individual. The capacity of an individual to adapt to challenges depends on their connections to other people and systems external to the individual through relationships and other processes.

For an individual person, resilience reflects all the adaptive capacity available at a given time in a given context that can be drawn upon to respond to current or future challenges facing the individual, through many different processes and connections. Resilience is not a trait, although individual differences in personality or cognitive skills clearly contribute to adaptive capacity. Supportive relationships play an enormous role in resilience across the lifespan [12,23,24]. Close attachment bonds with a caregiver and effective parenting protect a young child in multiple ways that are not located “in the child”.

Human individuals have so much capacity for adaptation to adversity in part because their resilience depends on many interacting systems that co-evolved in biological and cultural evolution, conferring adaptive advantages. Moreover, children are often protected by multiple “back-up” systems, particularly embedded in their relationships with other people in their homes and communities.

Resilience in a living, developing organism is both complex and dynamic—always changing because the individual and context are continually changing as a result of myriad interactions among people and their environments as well as ongoing interactions within a living person. Typically, we infer the presence of resilience from its manifestations in adaptive behavior or outcomes. It is theoretically possible, although it may be complex and difficult, to assess potential capacity for adapting to challenges prior to a demonstration of good adaptation to adversity. Yet practitioners often are called upon to make judgments about the capacity of a parent or a family or a community to handle a specific impending crisis. One of the ongoing challenges in resilience science is developing strategies of measurement that reflect the dynamic complexity of the concept.

## 2. Methods in Developmental Resilience Science

As investigators designed research, they had to operationalize their ideas about resilience. Initial studies often took the form of case reports or descriptions of people identified as high risk for problems who nevertheless appeared to be doing OK [12]. These individuals or groups were compared with others who shared similar risk profiles but were less successful by some criteria and/or others who shared similar successes but had lower risk histories. Typically, the goal was to identify the factors that might account for desirable adaptation in a context of adversity, the first clues to resilience. In the subsequent generation of research, the goal shifted to understanding how such factors might support health or positive outcomes, in a search for explanatory processes. Then, with strong evidence of processes that might contribute to resilience, investigators were able to test the promise of interventions to foster resilience in randomized controlled trials, gathering further evidence on the mutability of targeted processes. These three phases of research—first case studies, then studies of explanatory processes for positive outcomes, and third, intervention research—represent the first three waves of resilience science [2,6,12,25]. The current or fourth wave is focused again on processes of resilience, but it is far more integrative, as investigators attempt to understand resilience in multi-level, interconnected, and complex adaptive systems, from the molecular to the sociocultural level [15,20].

The fourth wave of resilience science required major advances in the tools available to measure multi-level processes and in the computational and statistical approaches for analyzing the data. Advances in these methods have spurred an exciting new era of research on resilience that ranges from epigenetic studies of parenting as a protective process to sociocultural influences on brain development [15].

Describing the many methods involved in resilience science is well beyond the scope of this commentary. Here we highlight the most fundamental questions and strategies that guided much of the research over the past five decades.

### 2.1. Three Core Questions Posed and Operationalized in Resilience Studies

Three basic questions have shaped the research on resilience. These core questions are presented in Table 1, along with examples of the constructs that were measured to address these questions. Resilience by definition relates to understanding desirable adaptation in the context of risk or adversity. Thus, two of the fundamental questions involve identifying the risks or adversities that are challenging the system and the criteria for evaluating how the system is faring or developing in the context of exposure to this kind of risk [26,27].

A wide range of adverse experiences have been studied under the rubric of resilience, including child neglect or abuse, separation and loss, family or neighborhood violence, terror or war, natural disasters, poverty, hospitalization, and many other kinds of adversity. Some studies focused on one kind of traumatic experience, such as rape or loss of a parent, while others assessed cumulative risk or multiple negative life events, now often described as “adverse childhood experiences” or ACEs [28,29]. Singular adversities, however, are rare, because the most severe forms of childhood adversity often reflect chronic, repeated, or combined exposures to traumatic events or hardships. As a result, many studies of resilience include indices of cumulative risk in some form.

Resilience research also requires a focus on constructs of adaptive function or development, the criteria by which adaptive success can be evaluated. Again, resilience studies have encompassed a wide range of criteria, including success in age-salient developmental tasks, such as learning to talk or read, behaving well in school, taking care of your children, or other expected achievements of individuals in a given historical and cultural context; emotional or physical health; and psychological well-being.

The third question addresses the central goal of resilience studies, which is to identify what makes a difference, the processes that account for how well individuals can meet the challenges (posed by adversities under study) to adapt (by the criteria under study). These are the promotive or protective factors that may explain how some people recover or thrive while others flounder or break down. These factors or processes can be studied at multiple levels of analysis, from the molecular or neurobiological to the cultural or societal level.

### 2.2. Promotive and Protective Factors

Resilience investigators often draw a distinction between promotive factors associated with generally better outcomes at any level of risk on the designated criteria of adaptive success and protective factors that play a special role in the context of high adversity or risk [15,30,31]. From a statistical perspective, promotive factors function as main effects where protective factors show moderating effects on risk or adversity, such that there is a larger effect when adversity levels are high than when they are low. Some of the most widely studied factors appear to have both effects, with effective parents, for example, promoting positive development across all levels of risk while also showing larger effects when conditions are more threatening [32]. Parents have multifaceted and versatile protective influences on many aspects of development, and it is not surprising they can step up their game when the well-being of their children is threatened by severe adversity.

### 2.3. Person-Focused and Variable-Focused Approaches

The initial waves of resilience science often included two major strategies of research: person-focused and variable-focused strategies [30,33] Person-focused methods include case studies and the identification of individuals within high-risk groups who show success in comparison with others who do not fare so well on the criteria for adaptive behavior. Studies of individual pathways of adaptive function also center on the person as a whole unit of interest. Variable-focused methods examine or test for linkages among the central variables of interest in resilience, including variables measuring risk or adversity, adaptive criteria of interest, and potential explanatory variables that may mediate, counter, or moderate the effects of risk or adversity on adaptive criteria. Some studies include multiple methods that combine person-oriented with variable-oriented strategies which offer complementary strengths [12,15,34,35,36]. Person-oriented methods recognize the wholistic function of a person as a living system whereas variable-oriented methods have advantages for delving more deeply into specific processes [15,30].

Contemporary multivariate strategies, such as growth mixture modeling, also represent a blended strategy because people are grouped by similarities in their intraindividual trajectories or patterns of change over time in one or more variables repeatedly measured over time to identify broader patterns of adaptive function encompassing multiple individuals [37]. These groups of people can then be compared on other variables.

The most powerful strategy for establishing the causal role of a hypothesized promotive or protective factor is a randomized controlled trial (RCT) that targets change in the purported causal factor and shows an effect of the intervention on the target and the outcome, mediated by change in the targeted process. Of course, this is feasible only when the purported buffer is malleable. Parenting skills provide a widely researched example in that they have been targeted in many randomized controlled trials aiming to boost parenting quality to promote resilience among children facing diverse adversities. RCTs on parenting interventions corroborate the extensive data suggesting that parenting quality is an important promoter and protector for child development [15,24,27,32,38].

## 3. Major Findings: What Matters?

An extensive literature has accumulated over the past five decades on resilience. Systematic reviews and meta-analyses of this literature have proven to be difficult due to variations in how resilience concepts are defined and operationalized, a point underscored early in this domain of science [39] but still relevant today [15,40]. Nevertheless, there has been a rather impressive convergence of findings across diverse literature from early days in resilience science. It is telling to compare classic early reviews by Garmezy [41] or Rutter [42] with recent reviews [12,15,24]. Many of the salient protective influences identified by clinical researchers pioneering this research domain remain salient today, although there have been major advances in technology for studying the processes involved at multiple levels of analysis, including epigenetic change, stress regulation, or dyadic interactions between parent and child.

### 3.1. Exposure Dose and Cumulative Risk

Dose matters. Many studies of resilience have shown that the severity of exposure either to one extremely traumatic event or in the sense of cumulative risk, makes a difference. Numerous studies utilizing different metrics of risk have shown a risk gradient effect where problems (of many kinds) are higher and adaptive success is lower in relation to the severity or accumulation of adversity experienced. Gradients are observed in studies of poverty, maltreatment, homelessness, war, natural disasters, ACEs, and other cumulative indices of exposure to risk [6,12,15,28,29,43,44,45,46].

At the same time, these studies also show variation among individuals at similar levels of risk, some of whom are manifesting positive adaptation and development consistent with the presence of considerable resilience. In effect, these adaptive individuals are “off the gradient” of risk, doing better than might be expected. These individuals motivated questions about how to account for their success and provided important clues for resilience science pertaining to the ongoing search for answers to the question of what makes a difference.

### 3.2. Promotive and Protective Influences: The Shortlist

Promotive and protective factors matter. The consistency of findings that emerged from this often-messy literature on the factors that seemed to matter for resilience among children at risk for diverse reasons suggested to the first author [6,12,30] that the phenomena under study were robust and widespread. Masten concluded that the “shortlist” of commonly observed protective factors in the literature reflected a set of fundamental adaptive systems that account for much of the capacity available to children for adapting to challenges as they grow up in families and communities. The adaptive processes reflected in the shortlist represent the legacy of biological and sociocultural co-evolution, selected over time for their adaptive value. They are common, nurtured in the normal course of development, and inherently renewable.

The original shortlist was focused on individual resilience factors commonly implicated in the literature [6,12,20,47]. More recently, Masten has compared the individual shortlist to common factors implicated in the surprisingly independent literature on family resilience; there are striking parallels [48] (Table 1, p. 19).

Table 2 shows an updated Shortlist based on the child literature with corresponding family resilience factors shown in parentheses.

This list represents a broad set of answers to the core question of resilience research: What makes a difference? The roles of these processes vary in salience over the course of development. Clearly, there are resilience factors, such as the belief that life has meaning, that are unimportant to infants who are instead depending on the quality of caregiving and the resilience of their families for their resilience to adversity.

Cultural variations infuse every aspect of the resilience shortlist. Cultural values influence family functions and practices as well as the expectations for child behavior and the ways that families socialize their children to fit into their culture, community, or society. However, unique culturally-based protective practices are not captured by the shortlist since it is based on common factors observed across different cultures. Interest and evidence of unique cultural traditions is growing as more investigators focus on the rich variation in cultural traditions around the world that may promote and protective human development in the context of adversity [49,50].

Each of the processes implicated by the shortlist could be studied across levels of analysis [20]. These are broad factors that undoubtedly engage and reflect many processes. As resilience science matures, we can anticipate more detailed elucidation of how these and other resilience factors work, from molecular to molar levels of biopsychosocial interaction.

### 3.3. Timing and Windows of Opportunity

Timing matters. The effects of adversity as well as promotive or protective influences vary by developmental timing [15,51]. Extensive evidence now documents the potential for long-term effects of “toxic stress” on development [52,53]. Early adversity can undermine brain development, caregiving quality, and other crucial aspects of experience for human development [54]. Growing evidence suggests that early adversity may have programming effects that influence later health and psychosocial development [15,55]. Adversity can undermine the development of key regulatory systems and fundamental protective systems for resilience. Chaotic environments, for example, may undermine the development of self-regulation skills essential for learning [56]. Postpartum depression may disrupt the formation of secure attachment bonds between a mother and baby while also interfering with the “serve and return” social interactions that play a central role in multiple aspects of early development [53].

At the same time, it is not the case that all forms of stress or challenge are “bad” for children. The development of adaptive skills and self-regulation systems appear to require experience with stress and challenges to optimize for an adaptive and healthy life, just as the immune system does [15]. Moreover, timing may matter. Growing up on a farm exposed to numerous microorganisms is protective for some allergies. Early exposure calibrates the immune system in ways that reduce later allergies, whereas later exposure to the same organisms can trigger an allergic reaction.

It has been recognized for decades in resilience research that some exposure to adversity can have beneficial effects. Early scholars of resilience described this nonlinearity in terms of “challenge” models [57] or “steeling effects” [42]. Animal models of stress inoculation suggest that experience with milder stressors can prepare an animal to handle major stressors more effectively [58,59]. Just as vaccinations are designed to boost immunity by exposure to manageable challenges, there is considerable interest in psychological inoculations to adversity achieved through exposure and some training in advance about what to do. School “lock-down” drills, a sad feature of modern childhoods in the age of terrorism, are a form of stress inoculation training.

Resilience studies also suggest that there are windows of opportunity for facilitating resilience through preventive interventions. Some of these windows reflect periods of plasticity in human development while others open as a result of adversity itself, customary transitions in a given society or culture, or when a combination of conditions converge for change. The preschool years, for example, are a period of rapid development of the neural systems, behavior, and social systems that support school readiness. Executive function (EF) skills advance quickly during this window, forecast early school success, and show considerable malleability in the context of preschool programs and other interventions [60,61]. Children from very high-risk backgrounds often lag behind in these skills, which are sensitive to adversities experienced by many of these children. As a result, investigative teams have developed interventions designed to promote EF skills as a strategy for promoting academic success in these children [62,63].

Resilience science also has implicated the transition years of late adolescence and early adulthood as a window of opportunity when “late bloomers” turn their lives in a more positive direction and young people who get off track during adolescence often find their way back to success [12]. This is a period of rapid brain development when executive control systems that support better planning and self-regulation are maturing. In addition, opportunities often open during this window as young people find mentors, join the military, go to college, become an apprentice, or fall in love. This confluence of opportunities, planning, support, and motivation set the conditions for positive change. Observations in the resilience literature of youth who turned their lives in a more positive direction during this period of life may be explained by this confluence of changing conditions.

## 4. Transformative Effects of Resilience Science on Practice Frameworks

The study of resilience in children was initiated by investigators with translational goals in mind. Parents, clinicians, and educators cannot wait for all the science to be completed. When a child needs help, adults in helping roles must act based on the best evidence available at the time [6,12,17]. From the early days of resilience science, many of these investigators worked to share their findings and perspectives to inform intervention and prevention efforts. Initially, resilience science shifted the conceptual frameworks of practice concerned with children at risk due to adverse childhood experiences. Ideas stemming from resilience frameworks originating in research have spread to many applied fields, with profound consequences for the models that guide intervention.

Resilience perspectives have had transformative effects on practice models in many pediatric professions concerned with child development, shifting models away from deficit-oriented approaches focused on risk and vulnerability to broader approaches that focus on positive goals, models, and measures, guided by strength-oriented philosophies [17]. This shift is evident in primary pediatric care [64,65,66,67,68] as well as many other fields of practice, including clinical psychology and psychiatry, school psychology and counseling, child welfare and social work, family therapy, services for military families, and humanitarian interventions around the world [15].

A resilience framework for intervention includes the following basic elements [17].

- Mission with positive goals
- Models and measures that include promotive and protective factors as well as positive criteria for evaluating success
- Methods to mitigate risk, boost assets, and mobilize adaptive systems
- Multi-sector and multi-level alignment to create synergy for change
- Maximizing leverage for change by strategic timing and targeting

Research on resilience in children suggests three basic strategies for intervention: reduce or mitigate risk; boost assets or reduce barriers to promotive factors for child health and development; and nurture, mobilize, or restore as needed the fundamental and powerful adaptive systems that generate capacity for resilience over the life course [6,12,17].

### 4.1. Risk-Focused Interventions

Fostering resilience does not mean that risk or adversity is ignored. Preventing or mitigating harm from adverse experiences is one of the most important approaches within a resilience framework. One of the most effective interventions of the 20th century was the widespread implementation of prenatal care to prevent premature birth.

Risk-focused interventions include strategies such as reducing the stress of pregnant women, screening and treating depression in mothers, reducing family violence and child maltreatment, or preventing homelessness. Many screening programs have been introduced successfully in primary care pediatric practice to identify well-established risk factors for children, both in the child and in the family situation, and refer families for intervention [5,69,70]. Efforts to integrate care for families may facilitate these efforts, including two-generation programs as well as efforts to integrate behavioral health care with primary health care; and embedding health care systems in natural habitats of children and their families, such as schools or community centers [71].

### 4.2. Asset-Focused Promotive Interventions

Promotive interventions focus on boosting the resources or assets available to child or family by adding resources or improving access to these promotive resources. Examples include provision of necessities, scholarships to attend a quality early childhood programs, cash transfer programs, programs to provide books or tutors, school buildings, libraries, playgrounds, and child-friendly urban environments. “Reach Out and Read” is an exemplary asset-focused strategy, a pediatric program of giving away books to young children when they come for routine check-ups to encourage reading aloud to children [72]. Similarly, a pilot program called “Sit Down and Play” provides children 2–24 months of age with a low-cost developmentally-appropriate toy during routine check-ups to promote child development through everyday play [73]. Some asset-focused programs are targeted to high-risk children: Minnesota, for example, funds early learning scholarships to low-income children to attend high quality early childhood programs. This program was motivated by compelling evidence of the high return on investing in early childhood, particularly for low-income children, including better health outcomes in adulthood, as well as better educational and employment outcomes and reduced risk for crime [54,74,75].

### 4.3. Protection-Focused Interventions to Engage or Mobilize Adaptive Systems

These interventions focus on nurturing, engaging, bolstering, or restoring powerful human adaptive systems that power major engines of protection in resilience. Efforts to improve parenting, provide an orphaned child with a new family, improve the quality of foster care, nurture brain development, provide social support, match youth to mentors, facilitate school engagement, or provide opportunities to develop skills and talents would be examples of this strategy. Many of the programs with the best evidence have focused on improving the quality of parenting or parent-child relationships in situations of risk or adversity, such as maltreatment, divorce, bereavement, foster care, or military deployment [32,38,76,77,78]. Programs to enhance individual-level factors such as self-efficacy or self-regulation also show promise for children and youth who have faced stress and trauma. Examples include recent pilot programs to promote positive sleep habits among children experiencing family homelessness [79] or to improve mindfulness skills among minority youth facing poverty [80].

## 5. Nurturing Resilience in Pediatric Systems

As evidence grows on the importance of childhood for lifelong health and well-being, so too does the significance of primary pediatric healthcare providers and other key adults in the normal lives of children for mitigating risk and supporting the development of resilience in children. We concur with the conclusion of Traub and Boynton-Jarrett, in *Pediatrics*, that “pediatricians are ideally situated to address trauma and build resilience” [5] (p. 10).

Traub and Boynton-Jarrett reviewed the state of resilience science, identifying what they describe as five modifiable resilience factors to improve child health outcomes in the near and far term: (1) positive appraisal styles and self-efficacy in children; (2) parenting; (3) maternal mental health; (4) self-care skills and household routines; and (5) trauma understanding. In their review, they make ten practical recommendations for pediatric practitioners which they describe as a blueprint for trauma-informed pediatric care [5] (p. 5).

### 5.1. Roles of Pediatric Healthcare Practitioners in Nurturing Lifelong Resilience

Pediatric practitioners and providers could nurture lifelong resilience in multiple ways. They are well-situated in the lives of families to educate parents about the importance of childhood for nurturing lifelong capacities for health and well-being. They can screen for promotive and protective factors and positive development in the lives of children as well as risks or problems. Many pediatric clinicians already screen for developmental milestones; adding checklists for assets and resilience factors in the lives of children would not be likely to overburden parents or practitioners while simultaneously conveying important public health messages. These messages can be tailored to age and context and referrals for normative developmental resources can be made available. Those resources could include programs such as Early Childhood Family Education, information on early childcare education quality ratings, or practice and provider resources from the American Academy of Pediatrics’ “Resilience Project”, including parent posters [81].

Widespread screening for assets and strengths in the lives of children and their families requires well-validated, easy to use tools which are not yet at hand. Traub and Boynton-Jarrett list four possibilities, scales developed in the 1990s and 2000s [5] (Table 4, p. 9). Most of these and other available measures are too long or too narrow in focus or age range. Most focus on individual factors to the neglect of relational and contextual strengths. Promising efforts to develop new tools are underway in an effect to capture in a usable format a broader array of individual, family, and community protective factors more consistent with the “shortlist” of resilience factors. One of the most widely used measures is the Child and Youth Resilience Measure (CRYM) developed by teams connected to the Resilience Research Centre in Halifax led by Ungar [82,83]. A short form of 12 items is available and forms of this measure have been utilized in multiple cultures and translated into multiple languages, including Arabic [84]. This measure includes items focused on individual, relational, and contextual resilience factors, although a version for parents to complete on younger children is not available. The Devereux Early Childhood Assessment—Clinical Form (DECA-C) is a parent-report measure for 2–5-year-olds that assesses three aspects of resilience: initiative, self-control, and attachment [85]. Narayan and Lieberman have developed a self-report measure of Benevolent Childhood Experiences (BCEs, pronounced “bee-ses”) completed by adults to capture positive experience in a format parallel to ACEs [28,86].

Child health professionals and the systems in which they practice offer ample opportunities to build child and family resilience. Increasingly, positive parenting is seen as a fulcrum within the context of child health and primary care [87,88]. Even relatively intensive parenting programs such as Triple P and the Incredible Years can be successfully adapted for delivery in such settings [89,90,91]. In a report detailing how strategies to promote parenting (and other factors influencing resilience) can be incorporated into child health care, the American Academy of Pediatrics (AAP) recommends that those working with children use an evidence-based strategy to communicate with families: offer help, express empathy, show loyalty, use families’ language about their concerns, partner with families to create individualized plans, and seek families’ permission when moving forward with plans [92]. A similar approach, motivational interviewing, is developmentally adaptable to help enhance resilience in children, adolescents, and parents [93]. For example, an evidence-based program that combines brief motivational approaches with parent training, the Family Check-Up, has been successfully delivered to families in clinics, schools, and community settings such as the Women’s Infants’ and Children’s Nutritional Program [94], with positive long-term effects that may be mediated in part through improved self-regulation [95]. Finally, child health professionals routinely influence resilience by interacting with other protective systems in children’s lives such as teachers [96].

### 5.2. Implications for Training

The most basic implication of resilience science for training is simply to ensure that practitioners in professions that routinely interact with children in roles related to health and human development are trained on the central concepts of resilience in development and evidence pertinent to their roles in practice on competence and resilience in development, as well as risks and vulnerabilities. There is currently a groundswell of training related to ACEs and trauma-informed care, which is important, but the other side of the story—the capacity of human individuals, families, and communities to overcome adversity also needs to be told. We also need “resilience-informed care”.

Effective clinicians in many fields, we believe, already utilize strengths-based models compatible with the resilience-oriented framework we suggest here. However, many of our healthcare systems of service delivery and training were built around “deficit” models that focused on risk, vulnerability, and pathology. Training professionals for assessment and interventions that encompasses positive health and development, promotive and protective influences, and systems beyond the individual child will require revamping training programs and professional development.

Recently, the AAP called for transforming child health professionals’ training to better promote children’s social-emotional health—their recommendations include stronger collaborations between developmental-behavioral pediatrics, child and adolescent psychiatry, primary care, and other child health professions, as well as integrated care models and innovative models of access [97]. Other professional organizations, such as the National Association of Pediatric Nurse Practitioners have made similar calls for training enhancements. The American Board of Pediatrics’ (ABP) annual report for 2017 highlighted the need to improve child health professionals’ skills with behavioral and mental health screening, prevention, and management, and the ABP continues to mobilize training efforts in this area [98]. In collaboration with the AAP and ABP, the National Academy of Medicine (NAM) developed ten training themes for the future child health workforce to advance children’s cognitive, affective, and behavioral development, including “recognize and mitigate risks for children’s healthy development” and “build on family strengths to promote wellness, resilience, and child care capacity”, [99]. Ongoing work in this area includes a 2018 NAM-ABP roundtable in which pediatric department chairs and residency program directors met to discuss how to best evolve training along these lines. One example of an innovative curriculum is “Tipping the Scales: The Resilience Game”, created by Harvard’s Center for the Developing Child, which is an interactive demonstration of how community-level factors and policy decisions influence outcomes for individuals and populations of children [100]. Another online training resource includes ways to strengthening families within the primary care medical home and video vignettes of patient/family encounters that show how to use motivational interviewing strategies to address common emotional-behavioral and mental health concerns in families [81].

## 6. Conclusions and Future Directions

Resilience science focused on human development continues to expand. Salient growth areas include the neuroscience of resilience, epigenetic processes, cultural processes, preventive interventions targeting protective processes, and humanitarian interventions in situations of mass trauma [6,12,15]. There is global interest in integrating sciences concerned with resilience to improve responses to large scale disasters, climate change, contagious diseases, war, and related crises [6]. Humanitarian agencies and governments are beginning to integrate their approaches and investments in human development, both horizontally across sectors (health, education, social services) and vertically across levels (child, family, community, society) [6,54].

Innovative strategies for assessing, monitoring, and supporting health, competence, and resilience are also emerging as technology advances. These include wearable devices, text messaging, social media, ecological momentary assessments, app-based programs, and virtual reality. Many of these methods have empowering potential for individuals and families to assess, monitor, and educate themselves and reach out to connect with social and professional support networks.

Formerly distinct lines of theory and research are coming together as the 4th wave of resilience science surges [6,12,15]. These include individual and family resilience studies; family and community resilience studies; resilience in childhood and aging; child development and economics; human resilience and organizational resilience; psychosocial resilience and ecological resilience. Five decades of research on human resilience have yielded important models for practice as well as theory, a host of methods, a robust list of protective factors, and many clues about future directions for research, given that the detailed processes involved in resilience require much further elucidation.

Professionals involved in child health care can play a central role in translating the science of resilience into the lived experience of children and their families, through their practice, training, and advocacy. Together with parents and teachers, they play profoundly important leadership roles in nurturing the systems that support healthy development across the life course and generate human capacity for resilience.

Going forward, a transdisciplinary, interprofessional framework is needed to mitigate risk and promote resilience in children. Effective preparation, practice, and policy for resilience requires collaboration and coordination across systems, with strategic consideration of the best targets, timing, and alignment of interventions. Many systems contribute to resilience as children develop and those children in turn enhance the future resilience of their communities and societies.

## Figures and Tables

**Table 1:** Core questions in resilience studies of individuals and examples of constructs measured.

What are the Challenges?	How Is the Person Doing?	What Processes Support Success?
*Risks*	*Criteria for Adaptive Success*	*Promotive or Protective*
Trauma	Developmental tasks	Neurobiological
Neglect	Mental health	Behavioral
ACEs ^1^	Physical health	Familial and relational
Poverty	Happiness	Community
Natural disaster	Work achievement	Cultural
War	Caregiving	Societal

^1^ Adverse childhood experiences.

**Table 2:** Shortlist of common resilience factors for child development.

Caring family, sensitive caregiving (nurturing family members)
Close relationships, emotional security, belonging (family cohesion, belonging)
Skilled parenting (skilled family management)
Agency, motivation to adapt (active coping, mastery)
Problem-solving skills, planning, executive function skills (collaborative problem-solving, family flexibility)
Self-regulation skills, emotion regulation (co-regulation, balancing family needs)
Self-efficacy, positive view of the self or identity (positive views of family and family identity)
Hope, faith, optimism (hope, faith, optimism, positive family outlook)
Meaning-making, belief life has meaning (coherence, family purpose, collective meaning-making)
Routines and rituals (family routines and rituals, family role organization)
Engagement in a well-functioning school
Connections with well-functioning communities

*Note*: Promotive/protective factors from the child literature are listed with corresponding family factors in parentheses.
